# Peritoneal inclusion cyst presenting as an umbilical hernia: case report and systematic review of the literature

**DOI:** 10.1093/jscr/rjae258

**Published:** 2024-05-03

**Authors:** Katie Nightingale, Emily Clough, Paul Goldsmith, Joshua Richard Burke

**Affiliations:** Department of Emergency General Surgery, Manchester University NHS Foundation Trust, Oxford Road, Manchester M139WL, United Kingdom; Department of Emergency General Surgery, Manchester University NHS Foundation Trust, Oxford Road, Manchester M139WL, United Kingdom; Department of Emergency General Surgery, Manchester University NHS Foundation Trust, Oxford Road, Manchester M139WL, United Kingdom; Department of Emergency General Surgery, Manchester University NHS Foundation Trust, Oxford Road, Manchester M139WL, United Kingdom; Leeds Institute Medical Research, University of Leeds, Beckett Street, Leeds, LS9 7TF, West Yorkshire, United Kingdom

**Keywords:** peritoneal inclusion cyst, multicystic peritoneal mesothelioma, umbilical hernia

## Abstract

Peritoneal inclusion cysts (PICs) are a rare and benign condition of uncertain pathogenesis. The fluid-filled, mesothelial-lined cysts manifest within the abdominopelvic cavity. This case report details an unusual occurrence of a 97 mm PIC- presenting as an umbilical hernia- in a 26-year-old male patient with no prior surgical history. Following pre-operative cross-sectional imaging, this was managed through open excision without complication. A systematic review of the literature highlighted 30 previous cases [26F, 4M] with a mean age of 34 years (std ±15.4) and a median diameter of 93 mm [IQR, 109 mm]. A total of 53% (n = 16) of cases had a history of previous abdominal surgery. Surgical excision is safe and laparoscopic modality should be considered (<1% recurrence). Accepting the limited evidence base, image guided drainage should be avoided (50% recurrence, n = 2).

## Introduction

Peritoneal inclusion cysts (PICs), also known as multicystic peritoneal mesotheliomas, are a rare and benign condition of uncertain pathogenesis [1]. The fluid-filled, mesothelial lined cysts manifest within the abdominopelvic cavity and can vary in size. In the few case reports presented, they are predominantly observed in women of childbearing age and have been associated with chronic peritoneal inflammation stemming from a variety of aetiologies and prior surgical interventions [2, 3]. To date, surgical excision has been the only considered definitive treatment [4]. This paper details a case of PIC presenting as an umbilical hernia, reported as per the for CAse REports (CARE) guidelines. [5], and a systematic review of the literature compliant with Preferred Reporting Items for Systematic Review Meta-Analysis (PRISMA) guidelines [6].

## Case report

**Figure 1:**
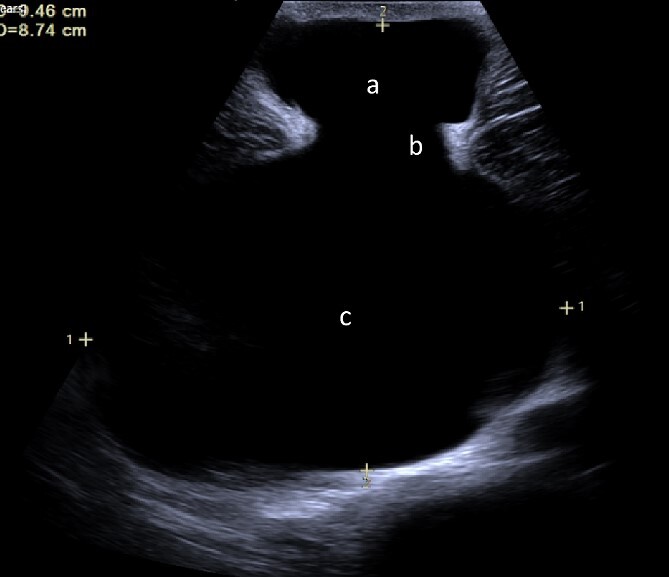
Ultrasound image demonstrating fluid collection within the umbilicus (a) tracking into abdominal cavity (c) originating from the cicatrix (b).

**Figure 2:**
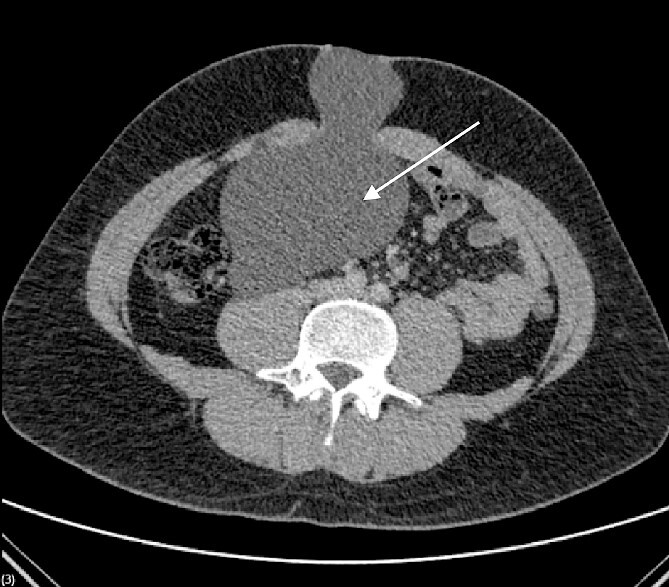
CT scan demonstrating bilobed cystic mass.

**Figure 3:**
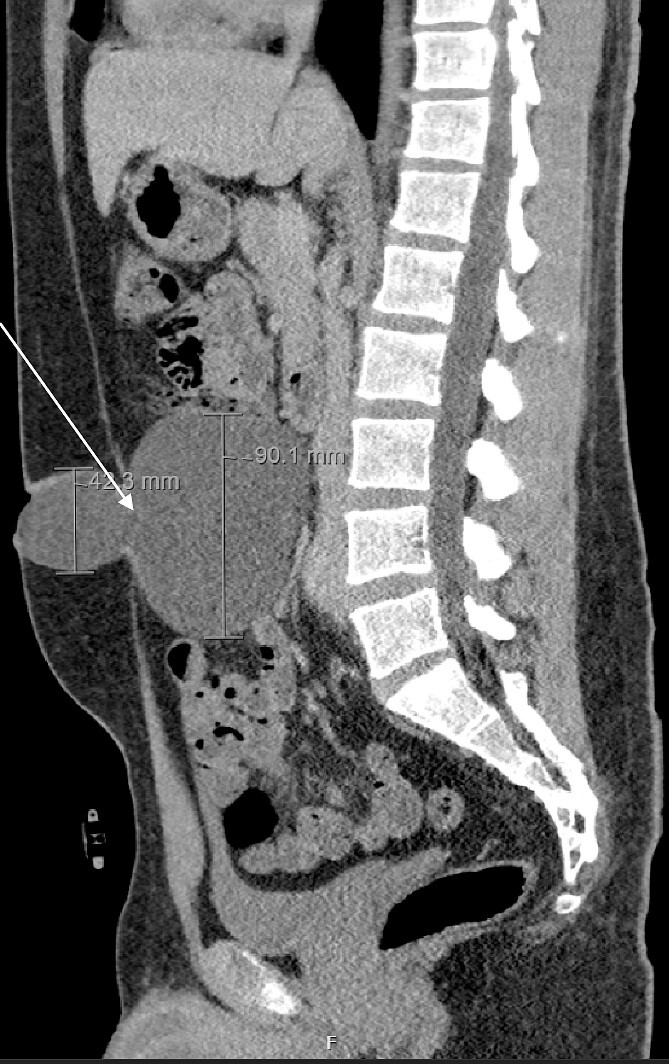
CT scan in sagittal cross-section demonstrating PIC.

**Figure 4:**
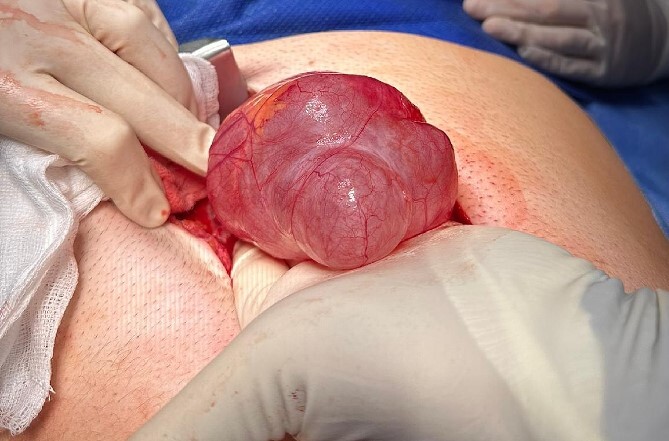
PIC was excised through midline laparotomy given the suggestion of retroperitoneal involvement on pre-operative imaging.

We present the case of a 26-year-old man who presented to the general surgery outpatient clinic with intermittent peri-umbilical pain, and the presence of a soft swelling palpable at his umbilicus. There was a positive cough impulse consistent with a true umbilical hernia and the patient described no previous medical or surgical history. An abdominal ultrasound scan visualized a large thin-walled serous fluid collection tracking into the peritoneal cavity (95 mm × 87 mm × 97 mm) (Fig. 1). Computed tomography (CT) of the abdomen and pelvis with contrast reported a large bi-lobed cystic mass centred at the right side of the mesentery, with herniation of part of the cyst along the umbilicus. Displacement of the small bowel with anterior extension into the abdominal wall was seen with the suggestion of posterior extension into the right retroperitoneal space (Figs 2 and 3). The patient underwent routine pre-operative work up and the cyst was excised through a midline para-umbilical laparotomy (Fig. 4) given the concern of retroperitoneal involvement. Intra-operatively, the hernia neck and the root of the cyst were found at the base of the umbilical cicatrix with no attachment to the mesentery. The cyst was loculated and filled with clear fluid. It was dissected off the peritoneal tissues and off from the posterior umbilical skin prior to removal. There was evidence of rupture of one of the locules and clear fluid was drained.

Histological examination demonstrated a paucicellular fibrous wall including frequent small blood vessels. The outer surface constitutionally comprised of loose fibrous tissue mixed with adipose tissue, and the lining was composed of a monolayer and- focally- a double layer of mesothelial cells. This showed no significant atypia, and a diagnosis of PIC with no evidence of neoplasia was made. He was discharged without complication, with a follow up CT planned at 6 months.

## Systematic search

**Figure 5:**
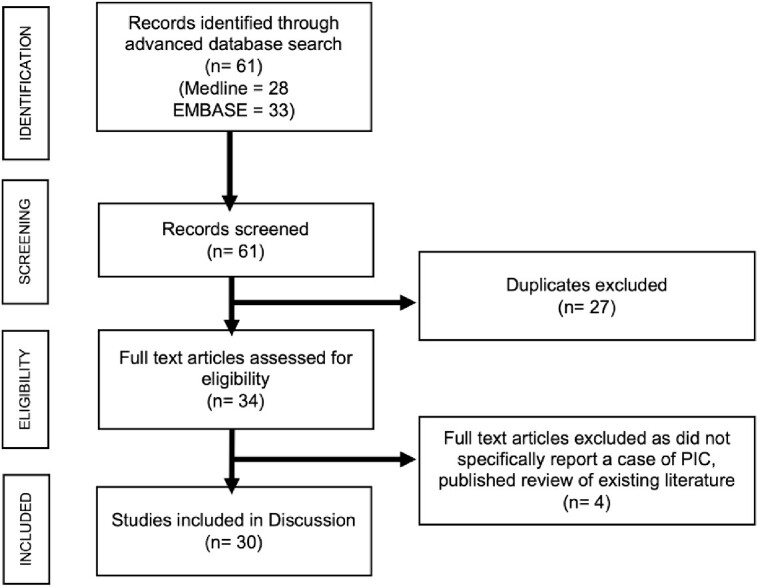
Preferred Reporting Items for Systematic Review Meta-Analysis (PRISMA) flow chart showing selection of reports for discussion.

The MEDLINE, EMBASE electronic databases published between 1 January 1947 and 1January 2024 were searched using the term ‘peritoneal inclusion cyst’. All studies which reported the case of a PIC were considered. KN independently retrieved article abstracts with JB cross-checking. The full texts of potentially eligible studies were retrieved and independently assessed for eligibility by KN and JB according to predefined inclusion criteria. Any disagreement was resolved through discussion with PG. Initial search yielded 61 full text articles from 1946 to 2023 for review as displayed in the PRISMA Flow Chart (Fig. 5), with 30 included in the final analysis (Table 1).

**Table 1:** Systematic search.

**Author(s)**	**Country**	**Sex**	**Age**	**Max.Diameter (mm)**	**Imaging**	**Origin**	**Intervention**	**Complications**	**Previous surgery**
Coulibaly *et al.* 2023 [7]	France	Female	29	-	PET	Pelvic cavity	Laparoscopic	None	Not documented
AlTamimi *et al.* 2023 [3]	United States	Female	18	150	CT	Ovary	Open	Failed IR drainage	Previous open bladder repair
Guo *et al.* 2023 [8]	England	Female	43	73	USS	Vaginal/uterine space	Open	None	Previous fibroid and ovarian cyst removal. Previous appendicectomy
Ivanova *et al.* 2022 [9]	Russian Federation	Female	-	-	-	Ovary	Laparoscopic	-	Not documented
Subramonian *et al.* 2021 [10]	Netherlands	Female	14	87	USS	Recto-vaginal space	Laparoscopic	None	Previous laparotomy (ileal perforation)
Cosgrove *et al.* 2021 [11]	Northern Ireland	Male	41	86	CT/MRI	Mesentery to the sigmoid colon	Open	None	Not documented
Katebi *et al.* 2021 [12]	Netherlands	Female	44	200	CT	Recto-vaginal space	Laparoscopic	None	Not documented
Kato *et al.* 2010 [13]	Japan	Female	21	-	Not recorded	Pelvic cavity	Not documented	Not recorded	Not documented
Wolf *et al.* 2020 [14]	Netherlands	Female	72	-	CT/MRI	Pelvic cavity	Open	Recurrence	Previous hysterectomy
Napoe and Rardin 2020 [15]	England	Female	47	50	CT	Uterine	Open	None	Hysterectomy
Katebi *et al.* 2019 [12]	Netherlands	Female	44	200	MRI	Recto-vaginal space	Laparoscopic	Not recorded	Not documented
Pereira 2019 [16]	England	Female	34	107	MRI	Pelvic cavity	Spontaneously ruptured	None	Previous robotic assisted endometrial resection, appendicectomy and adhesiolysis
Mishra *et al.* 2018 [17]	India	Male	43	210	USS/CT	Mesocolon	Laparoscopic	None	None
De Luca *et al.* 2017 [18]	Netherlands	Male	49	80	USS	Mesenteric root / precaval	Laparoscopic	None	Previous open appendicectomy
Sato *et al.* 2017 [19]	Ireland	Female	-	-	-	Ovary	Laparoscopic	-	2 prior laparotomies
Singh *et al.* 2015 [2]	India	Female	-	-	CT	Ovary	Open	None	Previous bilateral tubal ligation
Trehan. 2014 [20]	England	Female	51	57	MRI	Recto-vaginal space	Laparoscopic	None	Previous bilateral tubal ligation
Kanasugi *et al.* 2013 [21]	Japan	Female	31	-	MRI/USS	Pelvic cavity	Open	None	Not documented
Saxena *et al.* 2011 [22]	United States	Female	7	80	USS/MRI	Ascending colon mesentery	Laparoscopic	None	Not documented
Ho-Fung *et al.* 2011 [23]	United States	Female		-	-	Pelvic cavity	Cystocentesis	None	Not documented
Dillman and DiPietro 2009 [24]	Germany	Female	16	-	USS	Ovary	Laparoscopic	None	Multiple previous adhesiolysis
Vallerie *et al.* 2008 [25]	United States	Female	29	217	USS	Pelvic cavity	Cystocentesis	Recurrence	2 previous exploratory laparotomies
Advincula and Hernandez 2006 [26]	United States	Female	36	100	CT	Recto-vaginal space	Laparoscopic	None	Previous hysterectomy and exploratory laparotomy
Phupong *et al.* 2005 [27]	Germany	Female	33	61	USS	Ovary	Laparoscopic	None	Not documented
Nayak *et al.* 2005 [28]	India	Female	26	0	Found during caesarean	Pelvic cavity	Open	None	Previous caesarean section
Durak *et al.* 2005 [29]	United States	Male	32	140	CT	Pelvic cavity	Open		Not documented
Toprak *et al.* 2004 [30]	United States	Female	24	-	USS/CT/MRI	Adnexa	Open	None	None
Omeroglu and Husain. 2001 [31]	United States	Female	31	75	CT	Ovarian	Open	None	Previous myomectomy
Brustmann 2000 [32]	United States	Female	21	200	-	Mesentery of terminal ileum	Open	None	Not documented
Lamovec and Sinkovec 1996 [33]	England	Female	68	200	CT/MRI	Mesentry of descending colon	Open	None	Not documented

## Discussion

This is the first documented case of a PIC originating from the umbilical cicatrix. Thirty reported cases of PIC have been identified, 26 female and 4 male, with a mean age at presentation of 34 years (std ±15.4) and a median diameter of 93 mm [IQR, 109 mm]. A total of 53% (n = 16) of cases had a history of a previous abdominal surgery. Thirteen were excised via laparotomy, 13 were excised laparoscopically, 2 were drained under Interventional radiology guidance, 1 ruptured spontaneously with no further sequalae, and 1 case did not report their operative approach. Of the two cases that were drained, one recurred. Of those excised, only one recurred (median follow up 24 months (range 0–60 months)).

The most common organ of origin described was ovarian- in 23% of cases (N = 7), followed by the recto-vaginal space (17%, n = 5). In seven cases (23%) the origin was broadly defined as the pelvic cavity. Other origins included mesentery of small and large bowel, adnexa, and the vaginal-uterine space.

This case underscores the atypical presentation of a PIC in a surgically naïve male patient, emphasisizing the importance of pre-operative imaging in those presenting with atypical features of hernia; this instance, skin changes and discharge in the absence of previous abdominal surgery. Existing literature supports surgical excision rather than drainage given the risk of recurrence. Surveillance and required follow up specificities are currently unknown and further research is needed to determine this.
